# Pomegranate Wastes Are Rich in Bioactive Compounds with Potential Benefit on Human Health

**DOI:** 10.3390/molecules27175555

**Published:** 2022-08-29

**Authors:** Federica Marra, Beatrix Petrovicova, Francesco Canino, Angela Maffia, Carmelo Mallamaci, Adele Muscolo

**Affiliations:** Agriculture Department, Mediterranea University Feo di Vito, 89124 Reggio Calabria, Italy

**Keywords:** antioxidants, bioactive compounds, nutraceuticals, phenols, pomegranate peels

## Abstract

Pomegranate use is increasing worldwide, as it is considered a tasteful healthy food. It is mainly used as fruit, juice, and jam. The pomegranate peel represents about 40–50% of the total fruit weight and contains numerous and diverse bioactive substances. The aim of this research was to analyze the pomegranate peel chemical composition of Wonderful cultivated in Southern Italy and treated with an innovative physic dry concentration procedure in comparison with the peel composition of freeze-dried Wonderful cultivated in Southern Italy, freeze-dried Wonderful cultivated in South Africa, and freeze-dried pomegranate cultivated in India. The specific aim was to verify how much the growth area, cultivar type, and dry procedure influenced the chemical composition of the peels in terms of valuable bioactive compounds. Spectrophotometric and HPLC identification methods were used to detect antioxidants, antioxidant activities, and phenolic and flavonoid components. Results evidenced that in pomegranate peels of Wonderful cultivated in Calabria and dried with the innovative process, total phenolic substances, total flavonoids, vitamin C, vitamin E, and antioxidant activities were the highest. Great amounts of single phenolic acids and flavonoids were found in Calabrian Wonderful peels dried with the innovative process. Overall, it emerged that a great amount of bioactive and diverse compounds found in Calabrian Wonderful pomegranate peel comes from the niche pedoclimatic conditions, and the physic drying innovative methodology turned out to be an advantageous procedure to concentrate and conserve biocompounds.

## 1. Introduction

Pomegranate (*Punica granatum* L., Lythraceae) is a tree native to the Middle East, now cultivated worldwide, especially in Mediterranean countries, China, Southeast Asia, and other tropical or dry areas [1]. Except for its delightful taste, its peel, fresh seeds, juice and leaves hold a broad gamma of bioactive compounds (phenolics, flavonoids alkaloids, ellagic acid, punicalagin, anthocyanins, and tannins) with antioxidant [2], anti-inflammatory [3], antimicrobial [4], anticancer [5], anti-cardiovascular [6,7,8], and anti-infective [9] activities. As claimed by in vitro assays, commercial pomegranate juice has three-fold the antioxidant activity of red wine and green tea. In pomegranate anthocyanins predominate over tannins, explaining its high reducing activity. Cyanidin-3,5-*O*-diglucoside and pelargonidin-3,5-*O*-diglucoside are the most representative anthocyanins in the different genotypes of pomegranate. Due to its great contents of different phytochemicals with health-promoting effects [10,11], pomegranate fruit is considered the king of the super fruits group [12], and its extracts are also used by the pharmaceutical industry for creating supplements in capsules [13]. Pomegranate cultivation covers, worldwide, about 300,000 ha, with a production of 3 million tons, of which more than 76% is located in India, Iran, China, Turkey, and the USA. The 500 cultivars of pomegranate that have been identified have different physical–chemical characteristics and produce fruits that differ in the amount and types of bioactive compounds [14,15]. Fruits are of the best quality at a temperature of 38 °C under a dry climate; thus, the Mediterranean basin has the appropriate climatic conditions, representing an ideal area for high production of good-quality pomegranate fruits. Mediterranean pomegranates are mainly based on local cultivars, and their composition can differ from those of Eastern varieties, displaying a large variety of physical–chemical traits and distinct flavor profiles. Wonderful is the most widespread commercial pomegranate cultivar planted in Mediterranean countries and represents the industry standard variety. In the last few decades, around the world, there has been an increasing interest in the use of pomegranate and its parts, justified by an increasing demand from health care consumers and the pharmaceutical and cosmetic industries [13]. Generally, the edible part of pomegranate is directly consumed as food, or used for the preparation of juices, canned beverages, jams, and for the flavoring and coloring of drinks; conversely, pomegranate peel (approximatively 26–30% of the total fruit weight), which currently still represents a waste to be disposed of, is attracting the attention of the scientific community for its high content of phytochemicals that allow it to individuate as a new source of bioactive compounds, such as flavonoids, phenolic acids, and tannins with well-ascertained antioxidant capacity [16,17,18]. It has been reported that pomegranate by-products, and punicalagins in particular, decrease the level of fats in the blood and have anticancer, antiviral, and anti-inflammatory properties [15,19,20,21]. The scientific community was previously focused on the chemical characterization and health effects of pomegranate as a fruit or juice and only few studies were recently focused on the amount and composition of the bioactive compounds present in the pomegranate peel, which usually are a mixture, the synergistic effect of which can often cause different physiological responses acting on different organ targets contemporarily.

Based on the above considerations, the aim of the present study was to analyze the pomegranate peel composition of the variety Wonderful cultivated in Southern Italy and treated with an innovative system of dry concentration by the Gioia Succhi food industry. The specific aim was to verify if the growth area, cultivar type, and dry procedure influencing the chemical composition of the peels in terms of valuable bioactive compounds with beneficial effects in the prevention of numerous diseases or metabolic disorders. A comparison between the peel chemical composition of Wonderful cultivated in Southern Italy and treated with a spray-dry system and the peel chemical composition of freeze-dried Wonderful cultivated in Southern Italy, freeze-dried Wonderful cultivated in South Africa [15,22], and freeze-dried Kullu and Himachal [23,24] cultivated in India and already used in a pharmaceutical scope was carried out.

## 2. Materials and Methods

### 2.1. Chemicals

Metaphosphoric acid, 2,2-diphenyl-1-picrylhydrazyl (DPPH), NaOH, nitro-blue tetrazolium, dichlorophenol-indophenol (DCPID), 2,2′-azino-bis (3-ethylbenzothiazoline-6-sulfonic acid) di-ammonium salt (ABTS^•+^), 6-hydroxy-2,5,7,8-tetramethylchromane-2-carboxyl acid (Trolox), phenazine methosulphate, ethanol, gallic acid, ethylene-diamine-tetra acetic acid (EDTA), ferrozine, 2,4,6-tris (2-pyridyl)-s-triazine (TPTZ), and iron sulphate heptahydrate were purchased from Sigma Chemical Co. (St. Louis, MO, USA). HPLC-grade methanol and acetonitrile (Sigma Aldrich, St. Louis, MO, USA, 99.99%), acetone (Sigma Aldrich, 99.5%), deionized water, formic acid (Carlo Erba, 95%), and hydrochloric acid (Carlo Erba, 37%) were used for sample extraction and HPLC analysis. All chemical standards—gallic acid; protocatechuic acid; procyanidin 1 and 2; syringic acid; *p*, *m*, *o*-coumaric acids; pelargonidin; *trans*-cinnamic acid; bergamottin; cyanidine 3 *O*-glucoside; catechin; vanillic acid; epicatechin; delphinidin; *trans*-4-hydroxycinamic acid; sinapinic acid; 3-hydroxycinnamic acid; myricetin; luteolin; punicalagin; 2,5 dihydroxy benzoic acid; caffeic acid; ellagic acid; naringin; apigenin-7-neohesperosside; spiraeoside; quercetin; kaempferol; tocopherol; chlorogenic acid; vicenin 2; eriocitrine; rutin; vitexin; quercitin-3 beta-d-glucoside; ferulic acid; and apigenin were purchased from Sigma Aldrich, St. Louis, MO, USA. Other chemicals were of analytical grade and purchased from Carlo Erba Reagents s.r.l. (Milan, Italy).

### 2.2. Pomegranate Peel Preparation and Extraction

Fruits of the cultivar Wonderful grown in Calabria were washed and hand peeled. The peel of mature Wonderful fruits was dried in different ways: (1) with an innovative process by Gioia Succhi, a Calabrian food transformation industry, that used a physical spray-dried innovative process (PDS) that is held as an industry secret, and (2) stored a −80 °C for two days and then lyophilized with a freeze-dry system (Cheimika, SH Top, Pellezzano SA, Italy) at −56 °C for 96 h (FD). The dried peels were analyzed for chemical characteristics.

### 2.3. Sample Extract Preparation

The extracts were obtained using the method described in Muscolo et al. (2020) [25]. Briefly, lyophilized pomegranate peels were extracted at room temperature (22–25 °C) with continuous stirring for 90 min with 15 mL 95% ethanol. The samples were centrifuged (Unicen 21 RT167, Ortoalresa Inc., Madrid, Spain) at 2370× *g* (4000 rpm) for 15 min and the supernatants were filtered with 1 mm Whatman 185 filter paper (Merck, Darmstadt, Germany), evaporated to dryness in a rota-vapor (Diagonal condenser RE 400, Stuart Equipment, ST15, Stone, UK), and re-suspended in a final volume of 3.0 mL 95% ethanol. 

Lyophilized pomegranate peels were extracted at room temperature with continuous stirring for 60 min with 2.0 mL dH20 (Intercontinental Mod still 3/ES, Bioltecnical Service, s.n.c., Rome, Italy). The samples were then centrifuged at 590× *g* (2000 rpm) for 10 min and the supernatants were filtered with Whatman 1 filter paper and used for the determination of protein, carbohydrates, and ferrous chelating activity.

### 2.4. Determination of Total Phenolic Compounds, Total Flavonoids, and Vitamins A, C, and E in Pomegranate Peel

Total phenol content was determined with Folin–Ciocalteu reagent according to Muscolo et al. [25]. Briefly, 500 µL of the aqueous extract was mixed with 250 µL of Folin–Ciocalteu reagent and 2 mL of a 20% Na_2_CO_3_ aqueous solution, and the mixture was filled up to 50 mL with deionized water and placed in the dark for 1 h. The absorbance was measured at 765 nm using a UV-Vis Agilent 8453 spectrophotometer (Agilent Technologies, Agilent Technologies, Santa Clara, CA, USA). The results were expressed as mg/L of gallic acid equivalents.

Total flavonoid content was determined according to the colorimetric method as reported in Muscolo et al. [25]. The absorbance was measured at 510 nm using a UV-Vis Agilent 8453 spectrophotometer (Agilent Technologies, Agilent Technologies, Santa Clara, CA, USA). The results were expressed as rutin equivalents (mg/L) using a calibration curve.

Vitamin A was detected as reported in Aremu and Nweze [26]. Absorbance was read at 436 nm and vitamin A was expressed as retinol equivalent (RE).

For vitamin C (ascorbic acid) determination, the method of Davies and Masten [27] was used. Pomegranate powders (0.10 g) were extracted with 10 mL of 3% meta-phosphoric acid—98% acetic acid centrifuged at 2370× *g* (4000 rpm) for 10 min, and the supernatant was used for the determination of ascorbic acid.

For vitamin E (α-tocopherol) analysis, pomegranate powder (0.10 g) was extracted with 10 mL of hexane:isopropanol solution (3:2 *v*/*v*) with agitation for 5 h, and centrifuged at 1330× *g* (3000 rpm) for 10 min. The supernatant was used for the determination of vitamin E [28]. 

### 2.5. Protein and Carbohydrate Detection in Pomegranate Peel

Soluble protein was determined using the Bradford method as reported in Muscolo et al. [25] by using Coomassie Brilliant Blue G-250. The absorbance of each sample was measured at 595 nm using an 1800 UV-Vis spectrophotometer (Shimadzu, Kyoto, Japan). Bovine serum albumin > 99% purity (Sigma) was used as standard, and soluble proteins were estimated as mg BSA/g DW. 

The total available carbohydrates were measured using the anthrone method with minor modifications as reported in Muscolo et al. [25]. The amount of available carbohydrates was calculated using a glucose calibration curve (range of 10–100 mg/mL). The results were reported as mg/g DW.

### 2.6. Determination of Antioxidant Activities in Pomegranate Peel

The antioxidant activity against DPPH radical (2,2-diphenyl-1-picryl-hydrazyl-hydrate) was determined with the method reported in Muscolo et al. [25]. The DPPH concentration in the cuvette was chosen to give absorbance values of ∼1.0. Absorbance changes in the violet solution were recorded at 517 nm after 30 min of incubation at 37 °C. The inhibition I (%) of radical-scavenging activity was calculated as:

I (%) = [(A0 − AS)/A0] × 100(1)

where A0 is the absorbance of the control and AS is the absorbance of the sample after 30 min of incubation. Results were expressed as µmol Trolox/g DW.

The 2,2′-azino-bis-3-ethylbenzothiazoline-6-sulfonic acid assay (ABTS) was carried out according to Muscolo et al. [25] using a solution of 7 mM of ABTS in phosphate buffered saline (PBS). Aliquots of ethanol extracts (25, 50, and 100 μL) were added to 0.5 mL of ABTS^+•^ solution and brought to a final volume of 600 μL with PBS. After 6 min of incubation in the dark at room temperature the absorbance of the samples was measured at 734 nm. Results were expressed as µmol Trolox/g DW.

The total antioxidant capacity (TAC) was performed according to Muscolo et al. [25]. Sample absorbance was measured at 695 nm using UV-Vis spectrophotometer. Methanol (0.3 mL) in place of the extract was used as blank. The antioxidant activity was expressed as μg of α-tocopherol g^−1^ DW on a calibration curve.

### 2.7. RP-DAD-HPLC Identification of Phenolic and Flavonoid Components

Pomegranate peel was finely ground for analysis. By-product samples were subjected to solvent extraction before HPLC analysis for determination of the single phenolic and flavonoid compounds. Each sample was extracted in two different ways—0.1 g of previously lyophilised pomegranate peels was dissolved in 10 mL of 1% of HCl in methanol and 0.1 g of sample was dissolved in 10 mL of acetone solution: 1% of HCl in methanol (1:1). Each sample was analyzed in six independent replicates [25]. Reverse-phase–diode array detector–high-performance liquid chromatography (RP-DAD-HPLC) analyses of samples was carried out with a Shimadzu system (Kyoto, Japan), consisting of an LC-10AD pump system, a vacuum degasser, a quaternary solvent mixer, an SPD-M10A diode array detector, and a Rheodyne 7725i injector (Merck KGaA, Darmstadt, Germany). Separation of each compound was done on a 250 × 4.6 mm i.d. 5 μm Discovery C18 column supplied by Supelco Park (Bellefonte, PA, USA) and equipped with a 4.0 × 20 mm guard column. The column was placed in a column oven set at 25 °C. The injection loop was 20 μL and the flow rate was 1.0 mL/min. The mobile phase consisted of a linear gradient of solvent A (acetonitrile) in 2% acidified water (acetic acid:H_2_O, 2:98) as follows: 0–80% (0–55 min), 90% (55–70 min), 95% (70–80 min), 100% (80–90 min), and 0% (90–110 min). UV-Vis spectra were measured between 200 and 600 nm and simultaneous detection using a diode array at 278 and 325 nm. Compounds were measured using their retention time and UV spectra (Dueñas and Estrella, 2002), through comparison with purified standards (Sigma Chemical Co., Saint Louis, MO, USA).

### 2.8. Statistical Analysis

Analysis of variance was carried out for all the data sets. One-way ANOVA with Tukey’s Honestly Significant Difference tests were carried out to analyze the effects of treatment/cultivar on each of the various parameters measured. ANOVA and a *T*-test were carried out using SPSS software (IBM Corp. 2012, New York, NY, USA). Effects were significant at *p* ≤ 0.05. To explore relationships among different treatments/cultivars and chemical parameters, datasets were analyzed using principal component analysis (PCA).

## 3. Results and Discussion

**Figure 1:**
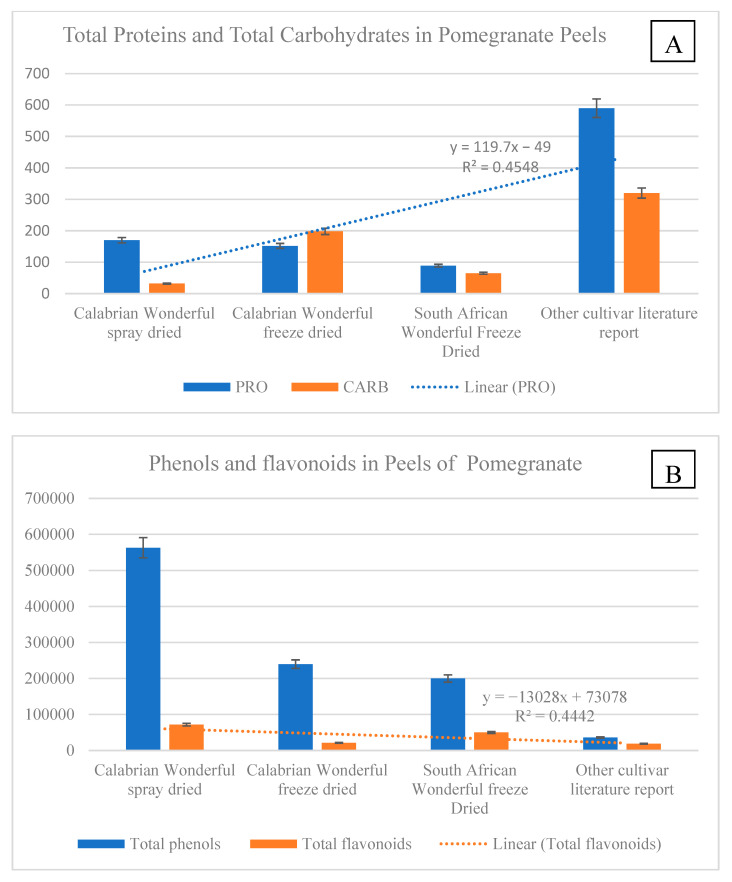
Soluble proteins and total carbohydrates (**A**) and total phenols and total flavonoids (**B**) in peels of different pomegranate cultivars dried differently. PSD (spray-dried Wonderful peel, experimental data); CFD (Calabrian Wonderful peel freeze-dried, experimental data); SAFD (South African Wonderful peel freeze-dried, literature data); IC (Indian cultivar peel freeze-dried, literature data). The experimental data are the mean of six replicates. Soluble protein (mg BSE g^−1^ DW), carbohydrates (mg g^−1^ DW), total phenols (µg TAE g^−1^ DW), total flavonoids (µg quercetin g^−1^ DW).

**Figure 2:**
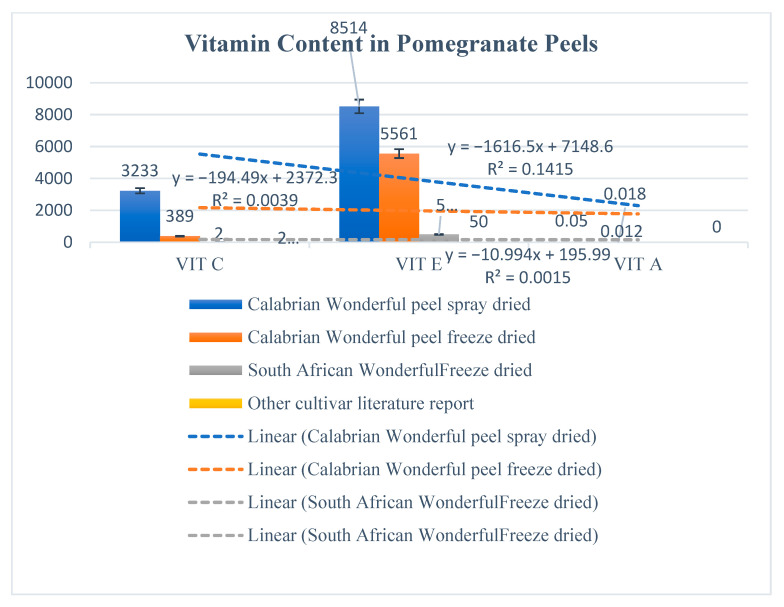
Vitamin A (µg retinol g^−1^ DW), C (µg ascorbate g^−1^ DW), and E (µg α-tocopherol g^−1^ DW) in peels of different pomegranate cultivars dried differently. PSD (spray-dried Wonderful peel, experimental data); CFD (Calabrian Wonderful peel freeze-dried, experimental data); SAFD (South African Wonderful peel freeze-dried, literature data); IC (Indian cultivar peel freeze-dried, literature data). The experimental data are the mean of six replicates.

**Figure 3:**
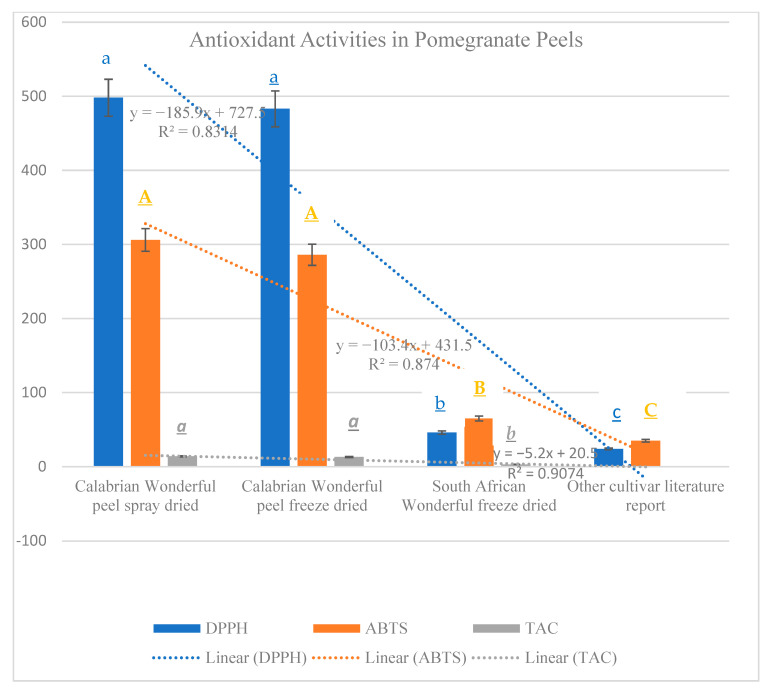
Antioxidant activities expressed as 2,2-diphenyl-1-picryl-hydrazyl-hydrate (DPPH), total antioxidant capacity (TAC), and 2,2′-azino-bis-3-ethylbenzothiazoline-6-sulfonic acid (ABTS) in peels of different pomegranate cultivars dried differently. PSD (spray-dried Wonderful peel, experimental data); CFD (Calabrian Wonderful peel freeze-dried, experimental data); SAFD (South African Wonderful peel freeze-dried, literature data); IC (Indian cultivar peel freeze-dried, literature data). The experimental data are the mean of six replicates. Different letters indicate significant differences *p* ≤ 0.05. Lowercase (DPPH), capital (ABTS), and italic (TAC).

Results evidenced that total carbohydrates were contained in a higher quantity in peels of other cultivars than in peels of Wonderful. The lowest carbohydrate content was found in spray-dried Wonderful peel (PSD) (Figure 1). Total proteins had an opposite trend, with the lowest in the peels of the other cultivars and the highest in PSD (Figure 1A). Total phenols and total flavonoids were present in the highest quantity in the Calabrian Wonderful spray-dried peels and in the lowest amount in the other cultivar peels. Total phenols were higher than total flavonoids in all the samples analyzed (Figure 1B). These data evidenced that the Wonderful cultivar had the majority of total phenols and flavonoids. These data highlighted that the geographic conditions, in which a determined type of cultivar grows, can drive the synthesis of bio-compounds, shifting the metabolism from primary to secondary. Data from Ramakrishna and Ravishankar [29] showed how drought conditions increased, in different plants and in different part of the plants, the amount of total flavonoids and phenolic acids that were used as antioxidants to overcome stress conditions. Vaneková et al. [30] showed how environmental factors such as altitude, habitat type, and sunlight exposure influenced the synthesis of total phenols and flavonoids in seven different cultivars of berries. The distribution of drylands is quite accentuated in southern and Mediterranean countries, and Calabria in particular is dominated by climate conditions mainly characterized by dry summers and mild wet winters that, as demonstrated by Fialho et al. [31], in pomegranates increased secondary metabolites with nutraceutical properties. The data of this research are in line with literature findings and highlight that the major quantity of total phenols and flavonoids contained in Wonderful peel cultivated in Calabria could be the result of the microclimatic conditions, which in turn affect metabolism, increasing antioxidants as well as antioxidant activities, and the soluble protein amount, which can have a double function of working as osmolytes or as antioxidative enzymes, as demonstrated by Kosová et al. [32]. In the spray-dried peel, the greatest amount of these compounds was found, evidencing that the innovative methodology used to dry the peels did not denature the bio-compounds but rather concentrated them. Vitamins were contained in greater amounts in the Wonderful cultivar than in the other cultivars, and were more concentrated in PSD and CFD. Vitamin E was the most abundant in all the pomegranate peel samples, except for the peel of the Indian cultivars. Vitamins have a great role as antioxidants and have important health benefits when consumed with the diet. The PSD contained a huge amount of vitamins (Figure 2). It was demonstrated that vitamins are enzymatic cofactors and act as antioxidants. Vitamin C increases under stress conditions to protect plants from oxidative stress by acting to detoxify reactive oxygen species (ROS) by direct scavenging or by acting as cofactors in the enzymatic reactions that involved ascorbate peroxidase and glutathione reductase enzymes [33]. Vitamin E, which is the most abundant vitamin, is a major single oxygen scavenger that provides protection against lipid peroxidation [34]. In support of the above findings, the activities of the antioxidant enzymes were greater in PSD than in the other samples. All the activities (DPPH, ABTS, and TAC) were expressed more in PSD and CFD than in the peels of the other samples (Figure 3). These data evidence that the innovative dry process did not affect the biological compounds and the enzymes. 

Single phenolic acids were higher in Wonderful peels than in the peels of the other cultivars. In PSD, the greatest amount of single phenolic acids was detected (Table 1). Ellagic acid was the most abundant, followed in ranking by 2-5 dihydroxy-benzoic, gallic, protocatechuic, *p*-coumaric, chlorogenic, and ferulic acids. It has been widely demonstrated that ellagic acid (EA) is a potent antioxidant with antimicrobial, anti-inflammatory, neuroprotective, antihepatotoxic, anticholestatic, antifibrogenic, anticarcinogenic, cytotoxic, and antiviral effects [35]. Recently, Reis Jordão et al. [36] evidenced that ellagic acid can be a promising alternative treatment for hypertension and cardiovascular disease, and Pei et al. [37] demonstrated that EA can be used to prevent diabetic cardiac dysfunction. The doses of EA generally tested in the prevention health treatments were 30 mg/kg. PSD peel contained a great amount of EA (240 mg/g), suggesting its possible use as a nutraceutical supplement for the prevention of numerous diseases. Additionally, gallic, 2-5 dihydroxy-benzoic, protocatechuic, and ferulic acids have been found in a number of phytomedicines with diverse biological and pharmacological activities, including radical scavenging, apoptosis of cancer cells, antihyperglycemic, antioxidant effects, and cardioprotective activity [38,39,40,41]. Among the single flavonoids (Table 2), procyanidin 2, punicalagin, procyanidin 1, and pelargonidin were, in this order, the most abundant compounds in PSD. Conversely, in the freeze-dried Calabrian and South African Wonderful peel, a lesser amount of single flavonoids than PSD was found, but in a greater quantity than the Indian cultivars. CFD had a greater amount of punicalagin (65 mg/g), procyanidin 1 (1.6 mg/g), procyanidin 2 (1.6 mg/g), and delphinidin than SAFD. IC contained a great amount of procyanidin 2 only in respect to CFD and SAFD.

Pearson’s correlation was used to determine the degree of correlation between selected reference data and variables (Table 3). As expected, TP was positively correlated with all the variables except for CARB. Vitamins and proteins were also positively correlated with all the variables except for carbohydrates. Total flavonoids did not correlate with the antioxidant activities and CARB. In addition, strong positive correlations between the antioxidant assays were reported at r = 0.998, *p* = 0.05, between DPPH and ABTS; at r = 0.996, *p* = 0.05, between DPPH and TAC; and at r = 0.988, *p* = 0.05, between ABTS and TAC (Table 3). This agrees with the results in Figure 1B suggesting that the high phenolic content in peel extracts determines the strong antioxidant activity.

**Figure 4:**
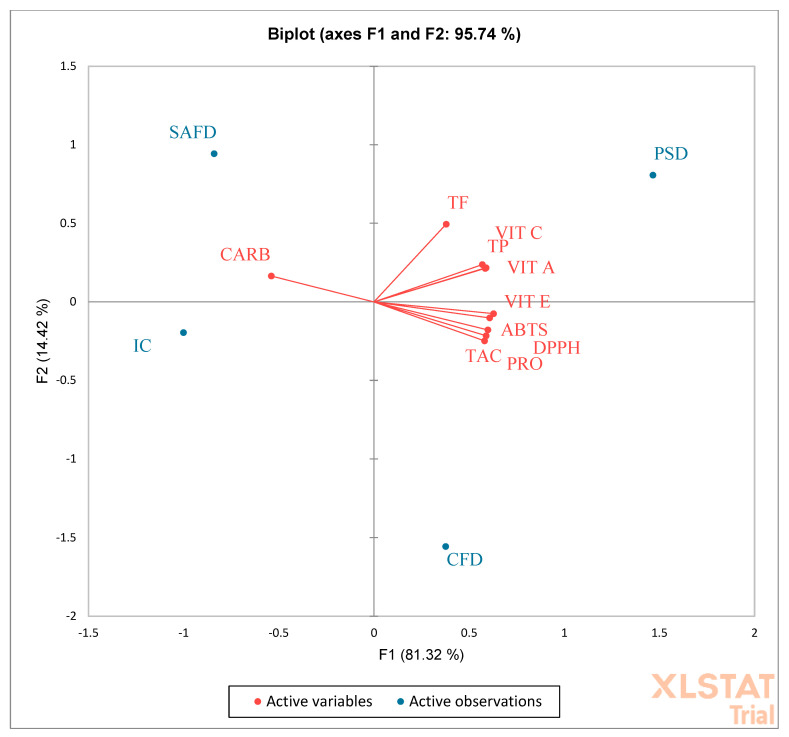
Total phenols (TP), total flavonoids (TF), proteins (PRO), vitamin C (VIT C), vitamin A (VIT A), vitamin E (VIT E), total carbohydrates (CARB), soluble proteins, 2,2-diphenyl-1-picryl-hydrazyl-hydrate (DPPH), total antioxidant capacity (TAC), and 2,2′-azino-bis-3-ethylbenzothiazoline-6-sulfonic acid (ABTS) contained in peels of different pomegranate cultivars dried differently. PSD (spray-dried Wonderful peel, experimental data); CFD (Calabrian Wonderful peel freeze-dried, experimental data); SAFD (South African Wonderful peel freeze-dried, literature data); IC (Indian cultivar peel freeze-dried, literature data).

**Figure 5:**
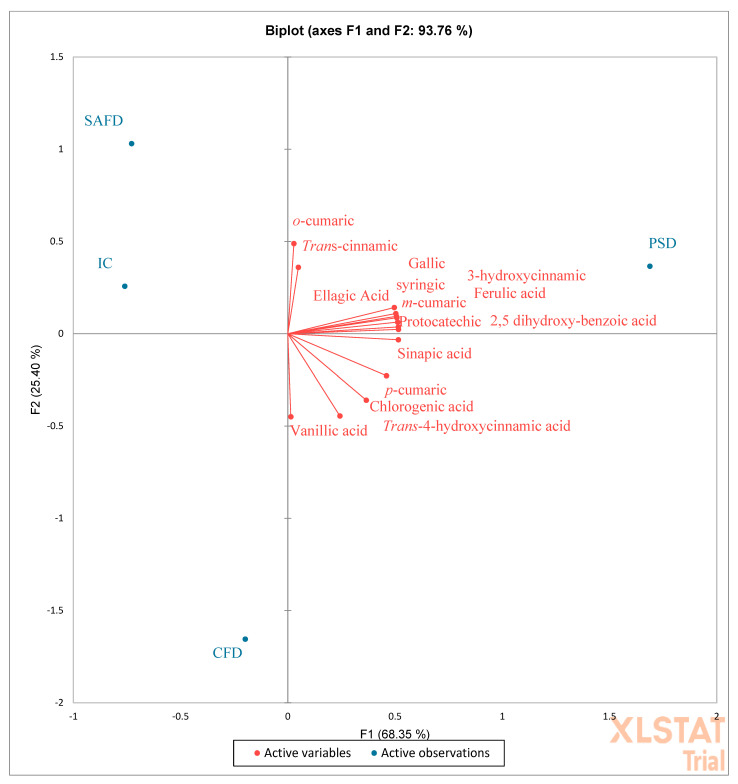
PCA (principal component analysis) diagram of single phenolic acids contained in the peels of different pomegranate cultivars dried differently. PSD (spray-dried Wonderful peel, experimental data); CFD (Calabrian Wonderful peel freeze-dried, experimental data); SAFD (South African Wonderful peel freeze-dried, literature data); IC (Indian cultivar peel freeze-dried, literature data).

**Figure 6:**
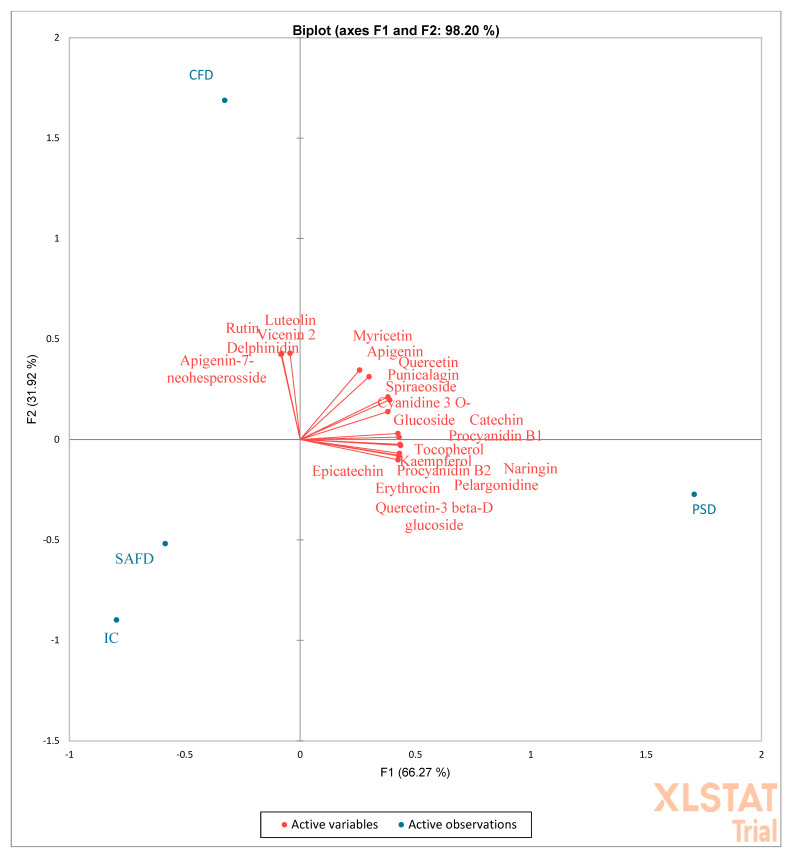
PCA (principal component analysis) diagram of single flavonoids contained in the peels of different pomegranate cultivars dried differently. PSD (spray-dried Wonderful peel, experimental data); CFD (Calabrian Wonderful peel freeze-dried, experimental data); SAFD (South African Wonderful peel freeze-dried, literature data); IC (Indian cultivar peel freeze-dried, literature data).

PCA analysis confirmed this assertion, and evidenced that TP, TF, VIT C, and VIT A were mainly correlated with PSD (Figure 4). No correlation between SAFD and IC was evidenced. Single phenolic acids correlated only with PSD (Figure 5), whereas single flavonoids were mainly correlated with PSD and in part with CFD. Rutin, luteolin, delphinidin, and apigenin were the single flavonoids in the highest quantities and correlated with CFD (Figure 6).

Procyanidins B1 and B2 were discovered to inhibit human colorectal adenocarcinoma and to improve the survival of chronic disease patients by reducing the complications of cardiovascular disease and metabolic syndrome, improving the overall quality of life [42]. Additionally, punicalagin is a flavonoid with proven antioxidant, hepatoprotective, anti-atherosclerotic, and antitumoral activity [43]. An ethical study was performed in 50 subjects (25 treated with supplements and 25 with placebo) to identify clinical features induced by 25 mg dried pomegranate (*Punica granatum*) fruit extract (which in turn contained 3.75 mg procyanidins) and 8.75 mg punicalagin–ellagic acid. Results evidenced after 60 days of treatment that the values for systemic oxidative stress, plasmatic antioxidant capacity, and skin antioxidant power increased significantly [44]. Other authors evidenced that the daily intake of pomegranate juice, rich in flavonoids and phenols, decreased the susceptibility of low-density lipoproteins (LDLs) to aggregate, and in cultured human coronary artery endothelial cells exposed to high shear stress, it down-regulated the expression of redox-sensitive genes and increased the functioning of blood endothelial cells [7]. Considering that peels contain many more phenols and flavonoids than juice, as already demonstrated by the previous study of Derakhshana et al. [45] and Russo et al. [46] carried out on different cultivars, it is possible to conclude that pomegranate peels represent a resource comparable to the fruit, if not better, that can be used as a source of bio-compounds with high added value in the nutraceutical field to formulate new supplements with beneficial effects on human health.

## 4. Conclusions

In short, the data evidenced that pomegranate peel is a valuable raw material rich in bioactive compounds. The amount and the type of bioactive compounds depends on the cultivars, but more so on the area where the plant grows.

Same cultivars grown in different conditions can have different amounts and varieties of phenolic compounds and diverse antioxidant activities, evidencing that the pedoclimatic variables drive the metabolism of the cultivars, increasing or decreasing specific secondary metabolites implicated in physiological adjustment of plants to adapt to stress conditions or changing conditions induced by climate. In addition, the spray-drying system developed by Gioia Succhi is innovative because it is able to perfectly concentrate and conserve the bioactive compounds with beneficial effects on human health in respect to the freeze-drying system, which has been already demonstrated by numerous studies to be much more relevant than the oven-drying procedure (40–60 °C). According to the achieved results, the high antioxidant capacity of pomegranate peel, exalted by the innovative method of drying, highlights its use as a supplement to preserve human health from various diseases. The innovative spray-drying method appears to be advantageous from an economic point of view, as it is able to condense the bioactive compounds considerably, providing a concentrated peel powder ready to be used in the nutraceutical field.

## Figures and Tables

**Table 1:** Single phenolic acids contained in the peels of different pomegranate cultivars dried differently. PSD (spray-dried Wonderful peel, experimental data); CFD (Calabrian Wonderful peel freeze-dried, experimental data); SAFD (South African Wonderful peel freeze-dried, literature data); IC (Indian cultivar peel freeze-dried, literature data). The experimental data are the mean of six replicates.

	PSD	CFD	SAFD	IC
	mg/g SS	mg/g SS	mg/g SS	mg/g SS
**Phenolic acids**				
Gallic	11.1 ^a^	0.2 ^c^	1.2 ^b^	0.2 ^c^
Protocatechuic	7.9 ^a^	1.4 ^b^	nd	nd
Syringic	0.6	nd	nd	nd
*p*-coumaric	5.8 ^a^	4 ^b^	0.09 ^c^	0.07 ^d^
*m*-coumaric	4 ^a^	0.6 ^b^	nd	nd
*o*-coumaric	0.8 ^ab^	nd	1.2 ^a^	0.5 ^b^
*Trans*-cinnamic	1.2 ^b^	0.4 ^c^	2.0 ^a^	0.3 ^c^
3-hydroxycinnamic	0.6	nd	nd	nd
*Trans*-4-hydroxycinnamic acid	0.6 ^b^	1 ^a^	0.3 ^b^	0.05 ^c^
Sinapic acid	0.7 ^a^	0.2 ^b^	0.05 ^c^	0.01 ^d^
2,5 dihydroxy-benzoic acid	16.4 ^a^	0.8 ^b^	0.4 ^b^	0.04 ^c^
Vanillic acid	0.8 ^b^	2.6 ^a^	0.8 ^b^	0.2 ^c^
Chlorogenic acid	5.6 ^a^	6 ^a^	1.4 ^b^	0.5 ^c^
Ferulic acid	4 ^a^	0.4 ^b^	0.3 ^b^	0.02 ^c^
Ellagic acid	240 ^a^	8 ^b^	2 ^c^	0.5 ^d^

Different letters in the same row indicate significant differences *p* ≤ 0.05.

**Table 2:** Single flavonoids in peels of different pomegranate cultivars dried differently. PSD (spray-dried Wonderful peel, experimental data); CFD (Calabrian Wonderful peel freeze-dried, experimental data); SAFD (South African Wonderful peel freeze-dried, literature data); IC (Indian cultivar peel freeze-dried, literature data). The experimental data are the mean of six replicates.

	PSD	CFD	SAFD	OCLR
	mg/g SS	mg/g SS	mg/g SS	mg/g SS
**Flavonoids**				
Procyanidin B2	178 ^a^	1.6 ^c^	1.2 ^d^	8.9 ^b^
Pelargonidin	5.8 ^a^	nd	nd	nd-
Cyanidin 3 *O*-glucoside	12.2 ^a^	4 ^b^	4 ^b^	0.15 ^c^
Catechin	12 ^a^	3 ^a^	0.03 ^c^	1.4 ^b^
Epicatechin	1.37 ^a^	nd	0.017 ^c^	0.07 ^b^
Delphinidin	0.8 ^b^	173 ^a^	0.39 ^c^	0.42 ^c^
Myricetin	1.2 ^a^	1.4 ^a^	nd	nd
Luteolin	nd	1	nd	0.0025
Naringin	0.9	nd	nd	nd
Apigenin-7-neohesperosside	0.2 ^b^	1.2 ^a^	0.34 ^b^	nd
Spiraeoside	1.0 ^a^	0.6 ^b^	0.5 ^b^	nd
Quercetin	3 ^a^	2 ^a^	0.3 ^b^	0.02 ^c^
Kaempferol	1.2 ^a^	0.2 ^b^	0.05 ^b^	0.1 ^b^
Procyanidin B1	13 ^a^	1.6 ^b^	1.2 ^b^	nd
Vicenin 2	nd	2	nd	nd
Rutin	0.3 ^b^	3 ^a^	0.56 ^b^	0.021 ^c^
Quercetin-3 beta-d glucoside	1.3 ^a^	nd	0.18 ^b^	0.05 ^c^
Apigenin	2 ^a^	2 ^a^	0.7 ^b^	0.037 ^c^
**Others**				
Erythrocin	0.9	nd	nd	nd
Punicalagin	86 ^a^	65 ^b^	40	28
Tocopherol	2.4	nd	nd	nd

Different letters in the same row indicate significant differences *p* ≤ 0.05.

**Table 3:** Pearson’s correlations (r) between total phenols (TP), total flavonoids (TF), vitamin A (VIT A), vitamin C (VIT C), vitamin E (VIT E), 2,2-diphenyl-1-picryl-hydrazyl-hydrate (DPPH), total antioxidant capacity (TAC), 2,2′-azino-bis-3-ethylbenzothiazoline-6-sulfonic acid (ABTS), total protein (PRO), and total carbohydrates (CARB). Values in bold are different from 0 with a significance level alpha = 0.05.

Variables	TP	TF	VIT A	VIT C	VIT E	DPPH	ABTS	TAC	PRO	CARB
TP	1	0.848	**0.954**	0.940	0.883	0.766	0.803	0.727	0.867	−0.649
TF	0.848	**1**	0.787	0.796	0.502	0.314	0.374	0.253	0.488	−0.232
VIT A	**0.954**	0.787	**1**	**0.998**	0.878	0.722	0.750	0.705	0.792	−0.783
VIT C	0.940	0.796	**0.998**	**1**	0.850	0.682	0.710	0.666	0.754	−0.770
VIT E	0.883	0.502	0.878	0.850	**1**	**0.964**	**0.972**	**0.958**	**0.972**	−0.880
DPPH	0.766	0.314	0.722	0.682	**0.964**	**1**	**0.998**	**0.996**	**0.979**	−0.825
ABTS	0.803	0.374	0.750	0.710	**0.972**	**0.998**	**1**	**0.988**	**0.990**	−0.809
TAC	0.727	0.253	0.705	0.666	**0.958**	**0.996**	**0.988**	**1**	**0.958**	−0.859
PRO	0.867	0.488	0.792	0.754	**0.972**	**0.979**	**0.990**	**0.958**	**1**	−0.760
CARB	−0.649	−0.232	−0.783	−0.770	−0.880	−0.825	−0.809	−0.859	−0.760	**1**

## Sample Availability

Samples of the compounds are available from the authors.
